# Bleeding and thrombosis in leukaemia patients admittet to an intensive care unit

**DOI:** 10.1186/2197-425X-3-S1-A252

**Published:** 2015-10-01

**Authors:** L Russell, M Madsen, J Bonde, A Perner

**Affiliations:** Rigshospitalet, Copenhagen University Hospital, Copenhagen, Denmark

## Intr

Mortality among leukemia patients admitted to the intensive care units remain high [1] and bleeding complications are common in these patients [2]. Prediction of bleeding would be of value because bleeding may associate with mortality. In recent years there has also been increasing focus on thrombosis among leukemia patients [3].

## Objectives

The basis for this study was a clinical observation of a high frequency of bleeding complications among ICU patients with leukaemia. We wanted to answer the following questions:

1. How big is the problem?

2. Can we predict bleeding or thrombosis using standard laboratory coagulation tests?

3. Is bleeding and/or thrombosis associated with a higher ICU-mortality?

## Methods

Retrospective observational study of patients with AML, ALL or MDS admitted to the ICU at Copenhagen University Hospital 2008-12. Data was collected from the ICU electronic patient files, the National Blood Bank as well as the hospital's clinical chemistry laboratory database. Bleeding complications were graded according to the WHO classification.

## Results

There was a high frequency of bleeding among these patients:

- 66 patients (57%) had one or several bleeding episodes during their ICU stay

- More than half of the bleedings were severe or debilitating (WHO grade 3-4)

- One third of all the admitted patients had at least one major bleeding episode during their ICU stay

- 10 deaths were directly related to bleeding episodes

- 11 patients (9%) had thrombi clinically or on ultrasound/CT-scan

Among severely thrombocytopenic patients (b-platelets < 50x10^9^/L) there was no difference among bleeders and non-bleeders with regard to platelet numbers, INR levels, activated pro-thrombin time, fibrinogen concentrations and anti-thrombin levels.

Higher 30-day mortality rates were observed in bleeding vs non-bleeding patients (68% vs 50%, p = 0.19.)

## Conclusions

Bleeding was very common and associated with mortality in leukaemia patients in the ICU, but it was difficult to predict in the individual patient using traditional coagulation analyses.
